# Thrombectomized autologous portal Y-graft inflow construction can be an option in living-donor liver transplantation: a case report

**DOI:** 10.1186/s40792-023-01641-8

**Published:** 2023-04-10

**Authors:** Munetoshi Akaoka, Koichiro Haruki, Kenei Furukawa, Shinji Onda, Shunta Ishizaki, Masashi Tsunematsu, Yoshihiro Shirai, Norimitsu Okui, Yoshiaki Tanji, Toru Ikegami

**Affiliations:** Division of Hepatobiliary and Pancreatic Surgery, Department of Surgery, The Jikei University School of Medicine, 3-25-8, Nishi-Shinbashi, Minato-Ku, Tokyo, 105-8461 Japan

**Keywords:** Living-donor liver transplantation, Portal vein thrombosis, Portal Y-graft interposition

## Abstract

**Background:**

In living-donor liver transplantation (LDLT), portal Y-graft interposition using the recipient’s portal vein (PV) bifurcation has been used for right lobe grafts with double PV orifices. We herein report the use of thrombectomized autologous portal Y-graft interposition for a recipient with preoperative portal vein thrombosis (PVT) in a right lobe LDLT with double PV orifices.

**Case presentation:**

The recipient was a 54-year-old male with end-stage liver disease due to alcoholic liver cirrhosis. There was PV thrombus in the recipient’s PV. The living liver donor was his 53-year-old spouse, and a right lobe graft was planned for the transplantation. Since the donor's liver had a type III PV anomaly, autologous portal Y-graft interposition after thrombectomy was planned for PV reconstruction in the LDLT. The portal Y-graft was resected from the recipient and a thrombus extending from the main PV to the right PV branch was removed on the back table. The portal Y-graft was anastomosed to the anterior and posterior portal branches of the right lobe graft. Followed by venous reconstruction, the Y-graft was anastomosed to the recipient’s main PV. The operation time was 545 min and the intraoperative blood loss was 1355 ml. The recipient was discharged on postoperative day 13 without any complications. The recipient remains well with the patency of the portal Y-graft one year after the liver transplantation.

**Conclusion:**

We herein report the successful use of autologous portal Y-graft interposition after thrombectomy on the back table for a recipient with PVT in a right lobe LDLT.

## Background

Portal vein (PV) reconstruction using a graft has been required for living-donor liver transplantation (LDLT) in the case of the right lobe graft with double PV orifices. Several methods have been proposed for the vascular reconstruction of the double PVs. Among them, portal Y-shaped graft interposition using the recipient’s PV bifurcation has been used worldwide due to its technical feasibility and good long-term patency [1, 2]. Although in the case of portal vein thrombosis (PVT), it is difficult to use a thrombosed recipient’s PV as a Y-graft, a study suggested advanced PVT can be removed by extensive thrombectomy [3]. We herein report the use of autologous portal Y-graft interposition after thrombectomy on the back table for a recipient with preoperative PVT in a right lobe LDLT with double PV orifices. To the best of our knowledge, this is the first report who underwent post-thrombectomy autologous portal Y-graft interposition in LDLT in the English literature.

## Case presentation

**Fig. 1:**
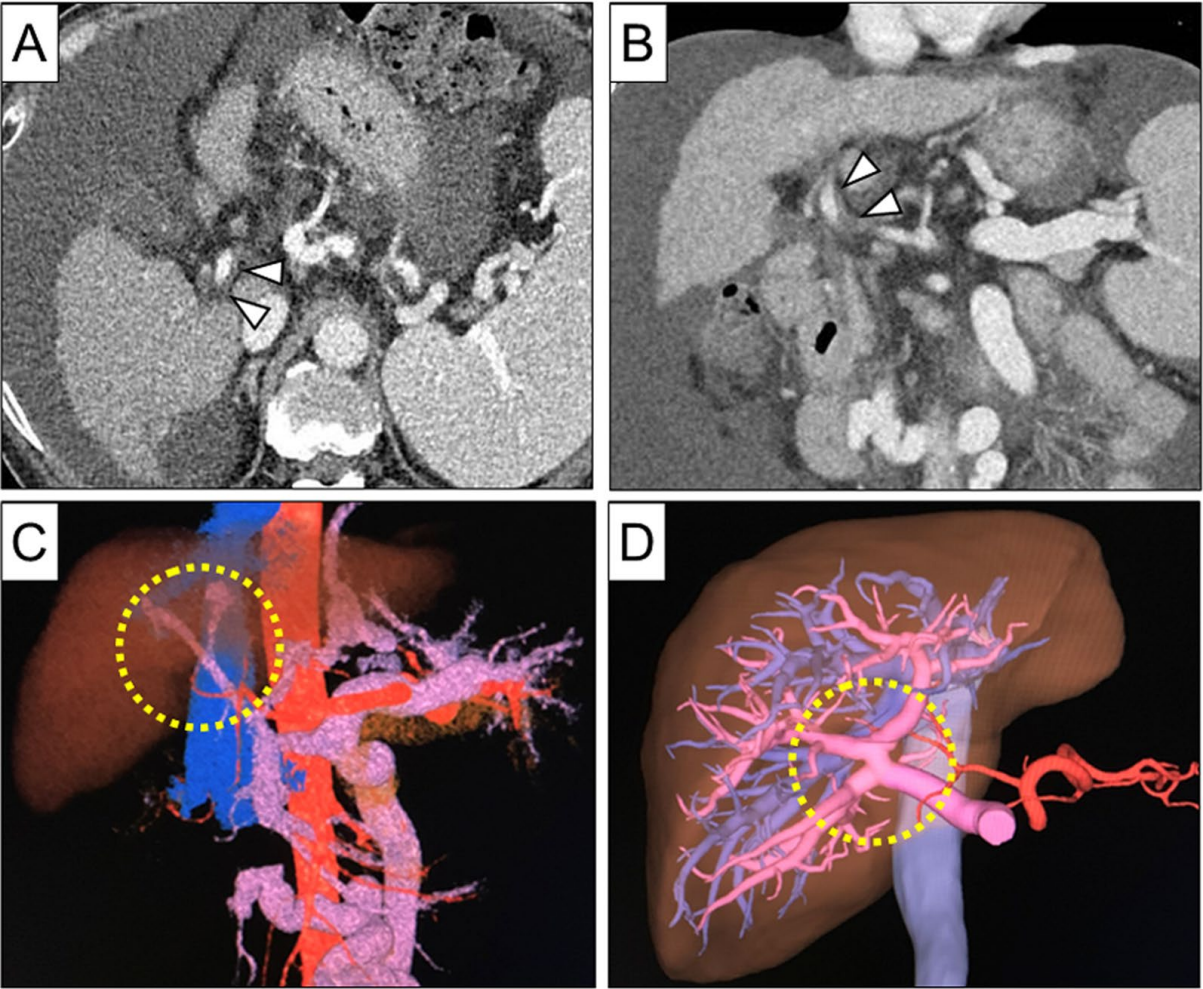
Preoperative imaging of recipient and donor. **A, B** Contrast-enhanced computed tomography showed a partially thrombosed main portal vein in the recipient (arrowheads). **C** Three-dimensional computed tomography showed the recipient’s portal vein which had standard anatomy and was narrowed by portal vein thrombosis (circle). **D** Three-dimensional computed tomography showed separating right anterior and right posterior portal veins in the donor (circle)

The recipient was a 54-year-old male patient with end-stage liver disease due to alcoholic liver cirrhosis. He had undergone radiofrequency ablation for hepatocellular carcinoma in segment 8, endoscopic variceal ligation for esophageal varices, and thrombolytic therapy for a PV thrombus extending from the superior mesenteric vein (SMV) to the right PV branch (Fig. 1A–C). Because of uncontrolled ascites, hepatic encephalopathy, and gastrointestinal bleeding caused by portal hypertension, LDLT was considered in the patient. The Child–Pugh score was 11 points (grade C) and the Model for End-Stage Liver Disease (MELD) score was 10. The recipient’s liver had standard PV anatomy (Fig. 1C), but there was PV thrombus remaining despite the thrombolytic therapy (Fig. 1A, B).

The living liver donor was his 53-year-old spouse with no pre-existing disease, and a right lobe graft was planned for the transplantation. The graft-to-recipient weight ratio (GRWR) calculated from computed tomography liver volumetry was 0.96. The estimated GWRW of the left lobe graft was 0.55, which did not meet our criteria of GRWR > 0.80 in our institution. The recipient was ABO blood type-incompatible, and thus preoperative rituximab and mycophenolate mofetil were administered according to the institutional protocol [4]. Since the donor’s liver had a type III PV anomaly (Fig. 1D), portal Y-graft interposition was planned for PV reconstruction in the LDLT. Although the recipient’s PV used as a Y-graft had a thrombus, a previous study suggested that the thrombus can be removed from the portal Y-graft on the back table [3]. Thus, the use of an autologous portal Y-graft was considered as an interposition graft.

**Fig. 2:**
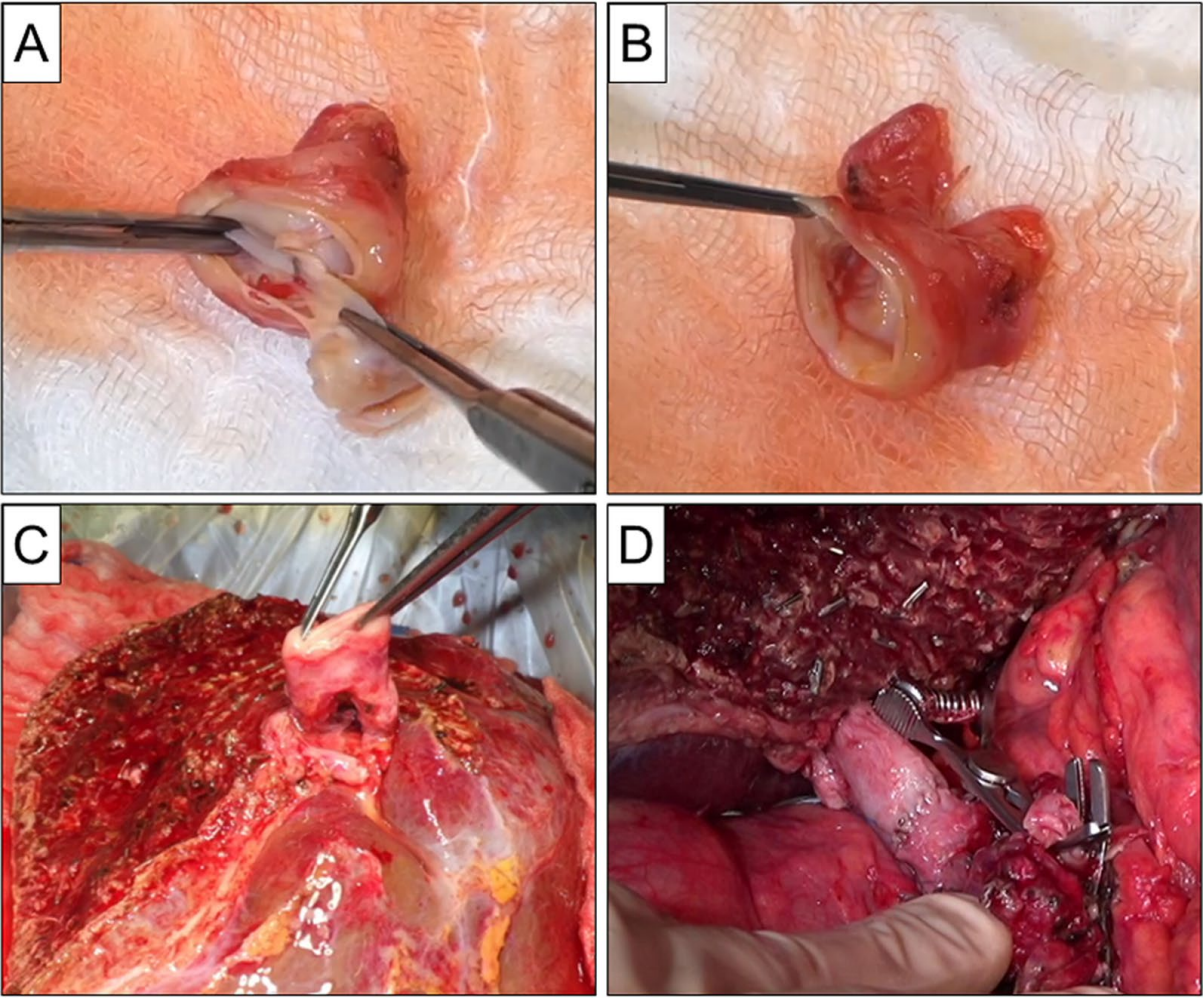
Autologous portal Y-graft interposition after thrombectomy. **A** Portal venous thrombus extending from the main portal vein (PV) to the right PV branch of resected portal Y-graft was easily removed on the back table. **B** Portal Y-graft after removal of thrombosis. There was no damage to the inner surface of portal Y-graft. **C** The right and left limbs of the portal Y-graft were anastomosed to the anterior and posterior portal branches of the right liver graft on the back table. **D** The main PV of the Y-graft was anastomosed to the remnant main PV of the recipient

In the donor surgery, in order to prevent iatrogenic injury to the donor's remnant PV, two right liver PV branches were cut separately. To use the thrombosed PV as autologous Y-graft interposition, after ligation of all the small branches from the hilar PV, the portal Y-graft was resected from the recipient. On the back table, a thrombus extending from the main PV to the right PV branch was easily removed without damaging the PV wall (Fig. 2A, B). Subsequently, the right and left limbs of the portal Y-graft were anastomosed to the anterior and posterior portal branches of the right lobe graft on the back table (Fig. 2C). The right hepatic vein and segment 8 vein (V8) were reconstructed to be a single orifice using the recipient’s left internal jugular vein [5]. Under cross-clamping of the inferior vena cava (IVC), the reconstructed liver venous orifice was anastomosed to the recipient’s IVC. The main PV of the Y-graft was then anastomosed to the remnant main PV of the recipient using a growth factor of 1 cm (Fig. 2D). In order to modulate the recipient’s portal inflow, the inferior mesenteric vein was ligated and the left gastric artery and vein were dissected using a surgical stapling device.

The operation time was 545 min and the intraoperative blood loss was 1355 ml. No anticoagulant was administered after PV reconstruction according to the protocol for the cases in which PV thrombectomy was performed [3]. The recipient and donor were discharged on postoperative days 13 and 9 respectively without any complications. The recipient remains well with the patency of the portal Y-graft one year after the liver transplantation.

## Discussion

In right lobe LDLT, some of the right lobe grafts with a type II PV anomaly and all of those with a type III PV anomaly have double PV orifices [1]. When the right lobe graft has double PV orifices, the best method of PV reconstruction has been under debate. There are several methods for the vascular reconstruction of the double PVs, such as Y-graft interposition, unification venoplasty, creation of a common orifice on the back table, and double anastomoses of two separate graft PV branches to the main PV of the recipient [6]. However, the use of autologous portal Y-graft has been accepted as a standard procedure because of its technical feasibility and good long-term outcome including better patency rate and less incidence rate of postoperative PVT [1, 2, 6]. When the recipient's PV is not available as a Y-graft owing to PV stenosis, the Y-shaped vein allograft or cryopreserved homograft can be used as an alternative [1]. Anastomotic stenoses and buckling deformities which may occasionally occur in reconstructed PVs in LDLT impair the PVs’ inflow, resulting in the need for surgical PV repair such as PV stenting [1, 2]. However, the Y-graft interposition, which consists of double anastomoses on the back table and subsequent single anastomosis to the main PV in the recipient, may provide a more natural alignment of the PV axes which can prevent buckling deformity of the PV after reconstruction.

It has been reported that 13% of recipients have PVT in LDLT [3]. The use of the thrombosed recipient's PV for PV anastomosis has been avoided and there has been no report showing that a thrombectomized PV can be used as an interposition Y-graft. A study suggested that extensive thrombectomy can be done under ultrasound guidance in adult LDLT with PVT [3]. Our strategy depends on the previous finding that there should be structured PVs in the cases of adult PVT and even massive thrombosis can be removed [3]. The feasibility of this procedure depends not on the degree of PVT but on the degree of atrophy of the recipient’s PV with PVT. Even if the recipient has severe PVT, the thrombus in PV can certainly be removed [3]. However, in recipients with a possible atrophic PV, surgeons must be ready for patch plasty of the native PV or the use of another interposition graft. Patients with adulthood-onset PVT have non-atrophic PVs because the establishment of PV structures has been completed during adolescence [3]. On the other hand, patients with childhood-onset PVT sometimes have atrophic PVs, some of which are unavailable for portal Y-graft interposition. Thrombectomy for PV thrombus has the risk of severe bleeding, however, thrombectomy on the back table can be performed without bleeding. Thus, our described method may be useful to PV reconstruction for grafts with double orifices in recipients with PVT. In order to avoid double PV orifices of the right lobe graft in LDLT, there is another method of discoid patch excision of the donor PV. The donor right PV branches are excised with a common discoid patch and anastomosed as a single opening. However, the defect in the donor’s remnant PV needs to be repaired, which leads to a risk of postoperative decreased portal inflow and PVT in the donor [6]. Therefore, when the right lobe graft has double PV orifices, the use of autologous portal Y-graft can be a better option, if available.

## Conclusion

We successfully performed autologous portal Y-graft interposition after thrombectomy on the back table for a recipient with PVT in a right lobe LDLT.

## Data Availability

All data generated or analyzed during this study are included in this published article.

## Abbreviations

GRWR: Graft-to-recipient weight ratio
IVC: Inferior vena cava
LDLT: Living-donor liver transplantation
MELD: Model for End-Stage Liver Disease
PV: Portal vein
PVT: Portal vein thrombosis
SMV: Superior mesenteric vein
